# Cardiac risk stratification in emergency resection for colonic tumours

**DOI:** 10.1093/bjsopen/zrab057

**Published:** 2021-07-06

**Authors:** G A Bass, M Forssten, A Pourlotfi, R Ahl Hulme, Y Cao, P Matthiessen, S Mohseni

**Affiliations:** 1 School of Medical Sciences, Orebro University, Orebro, Sweden; 2 Division of Traumatology, Surgical Critical Care and Emergency Surgery, University of Pennsylvania, Penn Presbyterian Medical Center, Philadelphia, Pennsylvania, USA; 3 Division of Trauma and Emergency Surgery, Department of Surgery, Orebro University Hospital, Orebro, Sweden; 4 Division of Surgery, Department of Clinical Science, Intervention and Technology, Karolinska Institutet, Stockholm, Sweden; 5 Division of Trauma and Emergency Surgery, Department of Surgery, Karolinska University Hospital, Stockholm, Sweden; 6 Department of Clinical Epidemiology and Biostatistics, School of Medical Sciences, Orebro University, Orebro, Sweden

## Abstract

**Background:**

Despite advances in perioperative care, the postoperative mortality rate after emergency oncological colonic resection remains high. Risk stratification may allow targeted perioperative optimization and cardiac risk stratification. This study aimed to test the hypothesis that the Revised Cardiac Risk Index (RCRI), a user-friendly tool, could identify patients who would benefit most from perioperative cardiac risk mitigation.

**Methods:**

Patients who underwent emergency resection for colonic cancer from 2007 to 2017 and registered in the Swedish Colorectal Cancer Registry (SCRCR) were analysed retrospectively. These patients were cross-referenced by social security number to the Swedish National Board of Health and Welfare data set, a government registry of mortality, and co-morbidity data. RCRI scores were calculated for each patient and correlated with 90-day postoperative mortality risk, using Poisson regression with robust error of variance.

**Results:**

Some 5703 patients met the study inclusion criteria. A linear increase in crude 90-day postoperative mortality was detected with increasing RCRI score (37.3 *versus* 11.3 per cent for RCRI 4 or more *versus* RCRI 1; *P* < 0.001). The adjusted 90-day all-cause mortality risk was also significantly increased (RCRI 4 or more *versus* RCRI 1: adjusted incidence rate ratio 2.07, 95 per cent c.i. 1.49 to 2.89; *P* < 0.001).

**Conclusion:**

This study documented an association between increasing cardiac risk and 90-day postoperative mortality. Those undergoing emergency colorectal surgery for cancer with a raised RCRI score should be considered high-risk patients who would most likely benefit from enhanced postoperative monitoring and critical care expertise.

## Teaser

Increased RCRI score is associated with increased mortality risk after emergency colonic cancer surgery.

## Introduction

Morbidity and mortality rates after emergency surgery for colonic cancer greatly exceed those after elective resections^1–6^. Large administrative data sets report postoperative complications in 27–44 per cent of patients following emergency colonic resection^1–4^. In several extensive studies, poorer survival outcomes were also identified in patients with colorectal cancer who initially presented as an emergency^5^^,^^7^. The physiological impact of emergency resection is mainly confined to the immediate postoperative period, and non-technical complications likely correlate with acute perturbation of the underlying medical pathology in these patients^8–11^.

Cardiovascular, respiratory, and cerebrovascular co-morbidities may delay a compromised patient’s recovery from postoperative complications, ultimately affecting overall outcome^12^. Perioperative risk mitigation, which is essential to optimal surgical care, is possible only when patients at risk are identified. Risk stratification tools, such as physiological predictors of critical care requirement (P-POSSUM score, Acute Physiology And Chronic Health Evaluation (APACHE) II, and Charlson Co-morbidity Index (CCI)) and surgical outcome (American College of Surgeons (ACS) Surgical Risk Score, National Emergency Laparotomy Audit (NELA) risk calculator, and the machine-learning Predictive OpTimal Trees in Emergency Surgery Risk (POTTER) score), assign a composite risk score to patients. However, their complexity limits their practical use in a time-sensitive clinical context^13–17^. The Revised Cardiac Risk Index (RCRI) estimates the risk of major postoperative cardiac complications or death for patients undergoing non-cardiac surgery^18–20^. Used efficiently at the point of care, the RCRI requires just six variables (high-risk type of surgery, ischaemic heart disease, congestive heart failure, cerebrovascular disease, diabetes requiring insulin, preoperative serum creatinine level over 2 mg/dl or renal insufficiency). Extensive validation of these tools across a range of non-cardiac surgical procedures has confirmed a positive correlation between increasing RCRI score and increased incidence of adverse outcomes^21–25^. Application of RCRI cardiac risk stratification to overall outcome after emergency oncological colonic resection has not been described in the literature. Postoperative risk-mitigation strategies, guided by simple tools such as the RCRI, may improve patient outcomes. Therefore, this study investigated whether RCRI predicted poor cardiac and overall outcomes following emergency cancer surgery for colonic cancer.

## Methods

Clinical, demographic, and oncological data on all adult patients who underwent emergency resection for colonic cancer between 1 January 2007 and 31 December 2017 were extracted from the Swedish Colorectal Cancer Registry (SCRCR) administrative data set^26^.

The SCRCR, a national database with over 99 per cent coverage, contains data on date of hospital admission, age, sex, AJCC TNM cancer stage, cancer location, ASA fitness classification, type of surgery, date of surgery, and hospital discharge date. Patients’ social security numbers were used to cross-reference the SCRCR data with date of death and known co-morbidities found in the Swedish National Board of Health and Welfare patient register. Co-morbidity data were used to calculate both the age-adjusted CCI and the RCRI^12^^,^^20^. The principles of the Declaration of Helsinki^27^ and STROBE guidelines (*Appendix S1*) were adhered to while designing and conducting this study, which received approval from the Swedish Ethical Review Authority (2020–05643). Patients were included if they were aged 18 years or older and underwent surgery classified as an emergency resection for colonic cancer. Patients were excluded if the tumour location was not specified, the operation date was missing, or the surgical procedure was recorded as transanal endoscopic microsurgery, local excision, or laparotomy without resection.

### Revised Cardiac Risk Index

The RCRI score was calculated, based on the preoperative presence of ischaemic heart disease, congestive heart failure, cerebrovascular disease, renal insufficiency, and diabetes mellitus diagnoses in the Swedish National Board of Health and Welfare patient register. Each variable scored 1 point if present. The original Cardiac Risk Index (CRI)^20^ used preoperative treatment with insulin for diabetic patients and a preoperative creatinine level exceeding 2 mg/dl. RCRI^28^, the simplified version of the CRI employed in the present study, uses the diagnoses of diabetes mellitus and renal insufficiency rather than laboratory values. The RCRI, validated elsewhere^22–25^, has supplanted the original CRI in clinical use.

### Data analysis and outcomes

Patients were divided into four cohorts with RCRI scores of 1, 2, 3, and 4 or more^20^^,^^28^^,^^29^. All patients received at least 1 point on the RCRI as emergency colonic surgery for cancer is considered an intervention with higher cardiac risk^29^. Clinical characteristics were summarized and compared between patients with RCRI 1 (no additional cardiac risk factors) and those with a calculated RCRI score of 2, 3, or at least 4. Data reviewed included age, sex, ASA classification, CCI score, cancer stage, type of surgery, co-morbidities, duration of hospital stay, and crude 90-day postoperative mortality. The primary outcome of interest was 90-day postoperative mortality. Secondary outcomes were 90-day postoperative mortality from a cardiac cause and cardiac morbidity.

### Statistical analysis

Categorical variables are reported as counts and percentages. Normally distributed continuous variables are shown as mean(s.d.) and those with a non-normal distribution as median (i.q.r.). To determine the statistical significance of differences between continuous variables, ANOVA was used if the data were normally distributed; otherwise, the Kruskal–Wallis *H* test was used. Pearson's χ^2^ test or Fisher’s exact test was used for analysis of categorical variables. A Poisson regression model was employed to determine the association between the RCRI and 90-day postoperative mortality, with adjustment for age, sex, neoadjuvant therapy, cancer stage, type of surgery, and co-morbidities not included in the RCRI. Results are presented as incidence rate ratios (IRRs) with 95 per cent confidence intervals. Two-sided *P* < 0.050 was considered statistically significant. Analyses were performed using R (R Foundation for Statistical Computing, Vienna, Austria).

## Results

Of 6392 patients registered as having undergone emergency resection for colonic cancer, a total of 5703 were included in this analysis. There was no statistically significant difference in type of surgery based on the RCRI score. Stage III colonic cancer was the most prevalent stage in all cohorts. Patients with higher RCRI scores were, however, less fit for surgery, and more likely to have an ASA grade of III or above (RCRI 4 or more *versus* RCRI 1: 90.0 *versus* 34.4 per cent; *P* < 0.001) and more overall co-morbidities with a CCI score of 7 or above (RCRI 4 or more *versus* RCRI 1: 90.0 *versus* 5.5 per cent; *P* = 0.002). These patients were also older (RCRI 4 or more *versus* RCRI 1: mean(s.d.) 79(8) *versus* 72(13) years; *P* < 0.001) and more often men (RCRI 4 or more *versus* RCRI 1: 55.5 *versus* 45.2 per cent; *P* < 0.001) (*Table 1*). Every registered co-morbidity increased in prevalence with higher RCRI scores, except for dementia, peptic ulcer disease, and liver disease (*Table 2*).

**Table 1:** Patient demographics

	RCRI 1 (*n* = 4401)	RCRI 2 (*n* = 911)	RCRI 3 (*n* = 281)	RCRI ≥ 4 (*n* = 110)	***P*** ^†^
Age (years)*	72(13)	77(10)	80(8)	79(8)	<0.001^‡^
**Sex ratio (M : F)**	2412 : 1989	441 : 470	116 : 165	49 : 61	<0.001
**ASA fitness grade**					<0.001
I	613 (13.9)	16 (1.8)	2 (0.7)	0 (0.0)	
II	2086 (47.4)	257 (28.2)	37 (13.2)	8 (7.3)	
III	1315 (29.9)	495 (54.3)	174 (61.9)	70 (63.6)	
IV	190 (4.3)	109 (12.0)	58 (20.6)	28 (25.5)	
V	7 (0.2)	2 (0.2)	1 (0.4)	1 (0.9)	
Missing	190 (4.3)	32 (3.5)	9 (3.2)	3 (2.7)	
**CCI score**					0.002
≤ 4	3452 (78.4)	268 (29.4)	11 (3.9)	1 (0.9)	
5–6	705 (16.0)	435 (47.7)	111 (39.5)	10 (9.1)	
≥ 7	244 (5.5)	208 (22.8)	159 (56.6)	99 (90.0)	
**Cancer stage**					0.014
I	140 (3.2)	29 (3.2)	14 (5.0)	4 (3.6)	
II	1314 (29.9)	280 (30.7)	82 (29.2)	27 (24.5)	
III	1575 (35.8)	353 (38.7)	120 (42.7)	45 (40.9)	
IV	1138 (25.9)	207 (22.7)	47 (16.7)	27 (24.5)	
Missing	234 (5.3)	42 (4.6)	18 (6.4)	7 (6.4)	
**Type of surgery**					0.080
Ileocaecal resection	188 (4.3)	52 (5.7)	13 (4.6)	9 (8.2)	
Right hemicolectomy	1970 (44.8)	422 (46.3)	147 (52.3)	60 (54.5)	
Transverse colonic resection	83 (1.9)	27 (3.0)	6 (2.1)	2 (1.8)	
Left hemicolectomy	515 (11.7)	109 (12.0)	23 (8.2)	15 (13.6)	
Sigmoid resection	704 (16.0)	129 (14.2)	38 (13.5)	10 (9.1)	
Anterior resection	52 (1.2)	10 (1.1)	4 (1.4)	0 (0)	
Hartmann’s operation	452 (10.3)	90 (9.9)	29 (10.3)	6 (5.5)	
Total colectomy	378 (8.6)	64 (7.0)	18 (6.4)	8 (7.3)	
Other operation	52 (1.2)	8 (0.9)	2 (0.7)	0 (0)	
Missing	7 (0.2)	0 (0)	1 (0.4)	0 (0)	

Values in parentheses are percentages unless indicated otherwise;

*values are mean(s.d.). RCRI, Revised Cardiac Risk Index; CCI, Charlson Co-morbidity Index.

† χ^2^ or Fisher’s exact test, except

‡ ANOVA.

**Table 2:** Co-morbidities

	RCRI 1 (*n* = 4401)	RCRI 2 (*n* = 911)	RCRI 3 (*n* = 281)	RCRI ≥ 4 (*n* = 110)	***P*** *
Myocardial infarction	0 (0)	146 (16.0)	127 (45.2)	89 (80.9)	<0.001
Congestive heart failure	0 (0)	150 (16.5)	134 (47.7)	91 (82.7)	<0.001
Peripheral vascular disease	79 (1.8)	67 (7.4)	34 (12.1)	22 (20.0)	<0.001
Cerebrovascular disease	0 (0)	264 (29.0)	118 (42.0)	57 (51.8)	<0.001
Dementia	99 (2.2)	47 (5.2)	10 (3.6)	4 (3.6)	<0.001
Chronic obstructive pulmonary disease	210 (4.8)	108 (11.9)	48 (17.1)	27 (24.5)	<0.001
Connective tissue disease	80 (1.8)	32 (3.5)	13 (4.6)	8 (7.3)	<0.001
Peptic ulcer disease	99 (2.2)	50 (5.5)	21 (7.5)	4 (3.6)	<0.001
Liver disease	36 (0.8)	11 (1.2)	2 (0.7)	2 (1.8)	0.320
Diabetes	0 (0)	310 (34.0)	148 (52.7)	73 (66.4)	<0.001
Hemiplegia	17 (0.4)	23 (2.5)	12 (4.3)	8 (7.3)	<0.001
Chronic kidney disease	0 (0)	41 (4.5)	35 (12.5)	40 (36.4)	<0.001

Values in parentheses are percentages. RCRI, Revised Cardiac Risk Index.

*χ^2^ or Fisher’s exact test.

An overall 90-day mortality rate of 13.5 per cent (770 deaths) was documented in the cohort. There was a statistically significant increase in crude 90-day postoperative mortality with increasing RCRI score (RCRI 4 or more *versus* RCRI 1: 37.3 *versus* 11.3 per cent; *P* < 0.001) (*Table 3*). After adjustment for clinically relevant co-variables, an RCRI score of 3 and at least 4 was significantly associated with increased postoperative mortality. Patients with an RCRI score of 3 had a 42 per cent increased incidence of 90-day postoperative mortality compared with those with RCRI 1 (adjusted IRR 1.42, 95 per cent c.i. 1.08 to 1.85; *P* = 0.012). An RCRI score of 4 or more was associated with a 107 per cent increase in the incidence of 90-day mortality (adjusted IRR 2.07, 1.49 to 2.89; *P* < 0.001) (*Table 4*).

**Table 3:** Crude outcomes

	RCRI 1 (*n* = 4401)	RCRI 2 (*n* = 911)	RCRI 3 (*n* = 281)	RCRI ≥ 4 (*n* = 110)	***P*** ^‡^
**Duration of hospital stay (days)** ^†^	9.0 (6.0–14.0)	11.0 (7.0–16.0)	11.5 (7.0–16.0)	11.0 (7.0–17.0)	<0.001^§^
Missing	55 (1.2)	13 (1.4)	1 (0.4)	1 (0.9)	
**90-day mortality**	499 (11.3)	162 (17.8)	68 (24.2)	41 (37.3)	<0.001
**Death after major cardiac event** ^*^	40 (8.0)	19 (11.7)	16 (24)	4 (10)	0.003

Values in parentheses are percentages unless indicated otherwise;

*percentage of all deaths;

† values are median (i.q.r.). RCRI, Revised Cardiac Risk Index.

‡ χ^2^ or Fisher’s exact test, except ^§^Kruskal–Wallis *H* test.

**Table 4:** Incidence rate ratio for 90-day mortality after emergency colonic cancer resection surgery

	**Incidence rate ratio**	*P*
**RCRI**		
1	1.00 (reference)	
2	1.12 (0.92, 1.35)	0.251
3	1.42 (1.08, 1.85)	0.012
≥ 4	2.07 (1.49, 2.89)	<0.001
**Age (per year)**	1.05 (1.05, 1.06)	<0.001
**Sex**		
F	1.00 (reference)	
M	1.1 (0.95, 1.28)	0.210
**Cancer stage**		
I	1.00 (reference)	
II	1.11 (0.69, 1.80)	0.698
III	1.23 (0.76, 1.98)	0.410
IV	2.74 (1.71, 4.39)	<0.001
**Type of surgery**		
Right hemicolectomy	1.00 (reference)	
Ileocaecal resection	1.36 (1.02, 1.80)	0.033
Transverse colonic resection	1.34 (0.9, 1.97)	0.146
Left hemicolectomy	0.71 (0.52, 0.97)	0.031
Sigmoid resection	0.86 (0.68, 1.1)	0.229
Anterior resection	0.68 (0.25, 1.81)	0.445
Hartmann’s operation	1.10 (0.86, 1.40)	0.454
Total colectomy	1.17 (0.90, 1.53)	0.234
Other operation	1.59 (0.94, 2.70)	0.085
**Peripheral vascular disease**		
No	1.00 (reference)	
Yes	1.09 (0.78, 1.52)	0.618
**Chronic obstructive pulmonary disease**		
No	1.00 (reference)	
Yes	1.30 (1.03, 1.64)	0.026
**Liver disease**		
No	1.00 (reference)	
Yes	1.30 (0.63, 2.71)	0.491
**Peptic ulcer disease**		
No	1.00 (reference)	
Yes	1.43 (1.03, 1.98)	0.030
**Dementia**		
No	1.00 (reference)	
Yes	1.66 (1.23, 2.24)	<0.001
**Connective tissue disease**		
No	1.00 (reference)	
Yes	1.50 (1.06, 2.12)	0.021

Values in parentheses are 95 per cent confidence intervals. The results are based on a Poisson regression model with robust standard errors. The model was adjusted for age, sex, cancer stage, neoadjuvant therapy, type of surgery, and co-morbidities. IRR, incidence rate ratio; RCRI, Revised Cardiac Risk Index.

## Discussion

Emergency surgery is associated with an increased risk of death compared with elective surgery, and this risk is further increased in the geriatric population^30^^,^^31^. In Sweden, the 30-day mortality rate after colonic cancer surgery, considered a disease of the elderly with a mean age of 73 years, is 7.5 per cent for emergency operations compared with 1.7 per cent for elective procedures^32^^,^^33^. One explanation for these findings could be that such patients are burdened by several co-morbidities, as demonstrated in the present study group, and to a larger extent frail, which can lead to less favourable outcomes after emergency surgery. Furthermore, preoperative optimization is time-sensitive; most patients present with obstruction or perforation due to cancer. The ensuing physiological derangements or sepsis put these patients at higher risk of postoperative adverse outcomes. Early identification of at-risk patients allows mobilization of resources and expertise in the perioperative phase that may abrogate this risk of adverse outcomes or failure-to-rescue events^34–37^. In addition to their validated applicability to clinical risk management, prognostic tools also facilitate the illustration of quantifiable risk to patients, empowering patient and family involvement and augmenting shared decision-making^38^–^40^.

Myocardial injury following non-cardiac surgery is negatively associated with 30-day surgical outcomes^41^. Patients with pre-existing ischaemic heart conditions are at particular risk of cardiovascular complications and death^28^^,^^42^. A linear relationship between increasing RCRI score and major cardiac events leading to in-hospital death after non-cardiac elective surgery has been confirmed previously^28^^,^^43–46^. The results reported here have demonstrated a robust linear association between increasing RCRI score and overall mortality after emergency cancer surgery for colonic cancer. Pre-existing cardiac co-morbidity, intuitively, was seen to be associated with excess morbidity and mortality in this population. Patients with an RCRI score of at least 4 (1.9 per cent of the total study cohort) were likely to be under-represented in this operative data set as a consequence of survival bias, whereby co-morbidity burden might have precluded proceeding to resection, or a more considerable proportion may have had laparotomy and a defunctioning stoma without resection during the initial surgical procedure, leaving tumour resection for a later stage. Although it was noted that patients with an RCRI score of 4 or more had the worst overall survival and cardiac-related mortality, their outcomes did not differ significantly from those with an RCRI score of 3 (4.9 per cent of the total study cohort).

The RCRI was chosen not just for its comparatively simple application at the point of care, but for its demonstrated prognostic superiority over other tools (including ASA grade and the original CRI) for the prediction of major cardiac complications^20^^,^^25^. Indeed, the RCRI was shown to achieve the same precision as the ACS National Surgical Quality Improvement Program Surgical Risk Calculator in predicting the risk of major adverse cardiac events, all-cause mortality, and morbidity in patients undergoing non-cardiac surgery^47^. Previous studies have assessed its external validity as a predictor of postoperative cardiac complications, cardiac death, and overall mortality after non-cardiac surgery. Perioperative risk mitigation, essential to optimal surgical care, is possible only when patients at risk are identified. However, most of these studies and meta-analyses pooled non-cardiac surgical specialties and procedures^25^. In the absence of specialty-specific granular data, applicability to date has been limited in the patient population of interest. However, the data analysis reported herein supports a strong association between the RCRI and early mortality after emergency colonic resection. The multivariable regression model demonstrated an increased 90-day postoperative mortality risk collinear with increasing RCRI score. Future study should compare the predictive accuracy and clinical value of the RCRI with those of other risk stratification tools, such as physiological predictors of critical care requirement (P-POSSUM score, APACHE-II, and CCI) and surgical outcome (ACS Surgical Risk Score, NELA risk calculator, and the machine-learning POTTER score).

The present study has several limitations. Although based on the validated SCRCR, it is subject to same inherent limitations as any retrospective analysis of an administrative database. The indication for emergency surgery as well as presenting physiological state of the patient were not captured. The results should be interpreted cautiously and in context as associative and hypothesis-generating, pending future research. A prospective longitudinal study would be required to demonstrate a causal relationship between outcomes and any cardioprotective therapies informed by preoperative risk stratification. The results indicate a strong association between increasing RCRI score and the risk of 90-day death; however, this study explicitly excluded all elective procedures, and interpretation of the data should be limited to emergency colonic cancer surgery. Furthermore, the database failed to capture patients deemed medically unfit for surgery, who were not offered tumour resection; it is likely that patients with an RCRI score of 4 or higher were preferentially excluded, mitigating the real effect of high RCRI score on survival. Although the RCRI may predict the risk of 90-day mortality in patients undergoing oncological colonic resection, other factors such as advanced age and frailty, advanced cancer stage, and the need for adjuvant chemotherapy are also likely to have some influence on 90-day survival. The regression model strongly associated advanced cancer stage and co-morbidities (such as chronic obstructive pulmonary disease, peptic ulcer disease, dementia or connective tissue disorder) with fatal outcomes. These results are expected and congruent with previous findings.

The RCRI is an easy and rapid predictive tool for risk stratification of patients undergoing elective colonic cancer surgery. Patients with a high RCRI score should be treated as high-risk patients who would most likely benefit from preoperative cardiac assessment and prehabilitation, as well as closer postoperative cardiac attention. Future studies should investigate whether such an approach improves survival outcomes.

*Disclosure.* The authors declare no conflict of interest.

## Supplementary material

Supplementary material is available at *BJS Open* online.

## Supplementary Material

zrab057_Supplementary_Data
